# Review of the epidemiology and variability of LRRK2 non-p.Gly2019Ser pathogenic mutations in Parkinson’s disease

**DOI:** 10.3389/fnins.2022.971270

**Published:** 2022-09-20

**Authors:** Paweł Turski, Iwona Chaberska, Piotr Szukało, Paulina Pyska, Łukasz Milanowski, Stanisław Szlufik, Monika Figura, Dorota Hoffman-Zacharska, Joanna Siuda, Dariusz Koziorowski

**Affiliations:** ^1^Department of Neurology, Faculty of Health Science, Medical University of Warsaw, Warsaw, Poland; ^2^Department of Medical Genetics, Institute of Mother and Child, Warsaw, Poland; ^3^Department of Neurology, Faculty of Medical Sciences in Katowice, Medical University of Silesia, Katowice, Poland

**Keywords:** LRRK2, Parkinson’s disease, epidemiology, pathogenic variants, p.Asn1437His

## Abstract

Parkinson’s disease (PD) is a heterogenous neurodegenerative disorder. Genetic factors play a significant role, especially in early onset and familial cases. Mutations are usually found in the *LRRK2* gene, but their importance varies. Some mutations, such as p.Arg1441Cys or other alterations in the 1441 codon, show clear correlation with PD, whereas others are risk factors found also in healthy populations or have neglectable consequences. They also exhibit various prevalence among different populations. The aim of this paper is to sum up the current knowledge regarding the epidemiology and pathogenicity of LRRK2 mutations, other than the well-established p.Gly2019Ser. We performed a review of the literature using PubMed database. 103 publications met our inclusion criteria. p.Arg1441Cys, p.Arg1441Gly, p.Arg1441His, p.Arg1441Ser are the most common pathogenic mutations in European populations, especially Hispanic. p.Asn1437His is pathogenic and occurs mostly in the Scandinavians. p.Asn1437Ser and p.Asn1437Asp have been reported in German and Chinese cohorts respectively. p.Ile2020Thr is a rare pathogenic mutation described only in a Japanese cohort. p.Met1869Thr has only been reported in Caucasians. p.Tyr1699Cys, p.Ile1122Val have only been found in one family each. p.Glu1874Ter has been described in just one patient. We found no references concerning mutation p.Gln416Ter. We also report the first case of a Polish PD family whose members carried p.Asn1437His.

## Introduction

Parkinson’s Disease (PD) is the second most common neurodegenerative disorder (Balestrino and Schapira, 2020). It is mostly sporadic disease, but about 15% of patients have monogenic mutations (OMIM; 168600). The genetic forms are usually found in early onset and familial cases (Balestrino and Schapira, 2020). Mutations in the *LRRK2* gene (OMIM; 609007), encoding dardarin – leucine-rich repeat kinase 2 (LRRK2), are the most commonly reported genetic causes of late-onset autosomal dominant PD-8 (PARK8; OMIM 607060). The LRRK2 belongs to the ROCO protein family and contains several protein–protein interaction domains and a catalytic core (a serine/threonine kinase domain) (Myasnikov et al., 2021). It is responsible for transmitting signals or helping to assemble the cell’s structural framework.

**FIGURE 1:**
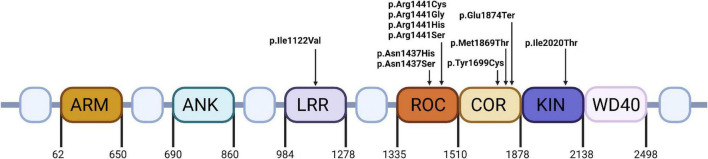
The structure and mutations’ localization of LRRK2. ARM, Armadillo repeat; ANK, Ankyrin repeat; LRR, Leucine-rich repeat; ROC, Ras-of-complex proteins GTPase; COR, C-terminal of ROC; KIN, kinase; WD-40, Trp-Asp-40.

**FIGURE 2:**
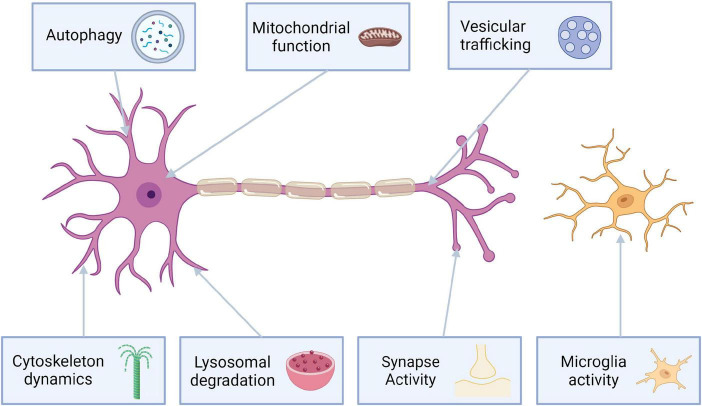
LRRK2 protein’s role in neurophysiology.

The pathophysiology of LRRK2 mutations is very complex. Most of the pathogenic mutations are located in either ROC-COR domain or kinase domain (Figure 1). Mutations associated with ROC-COR domain cause reduced GTP-ase activity, which in turn elevates kinase activity (Aasly et al., 2010). Kinase domain variants associated with PARK8 result in hyperactivation of kinase activity (Greggio et al., 2006). Proteins of Rab GTPase family are both a regulator and kinase substrate for LRRK2. They are responsible for intracellular vesicle trafficking, and improper vesicle trafficking has been reported in cells harboring PD-causative LRRK2 mutations. Rab proteins interact with LRRK2 to import it to lysosomes, or the trans-Golgi network to initiate serine/threonine kinase activity 2. Mutated LRRK2 affects the mitophagy process leading to mitochondrial depolarization (Papkovskaia et al., 2012). Additionally mutations in LRRK2, also lead to neuronal toxicity, reduction in neurite length and increased α-synuclein propagation (Shani et al., 2019; Figure 2).

The most common mutation in LRRK2 is p.Gly2019Ser substitution. It can be found in 30% Arab-Berber and 13% of Ashkenazi Jewish familial cases of PD (Lesage et al., 2006; Ozelius et al., 2006). It has also been reported in up to 6% of familial and 2% of sporadic European PD cases (Lesage et al., 2005). In the Polish population LRRK2 p.Gly2019Ser is rare (Milanowski et al., 2021). However, this mutation has very reduced penetrance which increases with age. It is thought that approximately 25–42.5% of p.Gly2019Ser carriers will develop PD before the age of 80, as penetrance of this mutation depends on a polygenic risk score (Healy et al., 2008; Lee et al., 2017). Other mutations are less commonly found and are usually reported in only a few families. Contrary to p.Gly2019Ser mutation, with its reduced penetrance, other pathogenic mutations were not found in healthy controls (excluding rare cases of p.Arg1437 carriers). This may suggest that those mutations express a higher pathogenic effect compared to p.Gly2019Ser. However due to the small number of analyzed patients this thesis remains unclear.

The aim of our study is to report the first Polish family with the LRRK2 p.Asn1437His mutation. We also want to summarize and review the current knowledge regarding LRRK2 non-p.Gly2019Ser pathogenic mutations.

## Materials and methods

**FIGURE 3:**
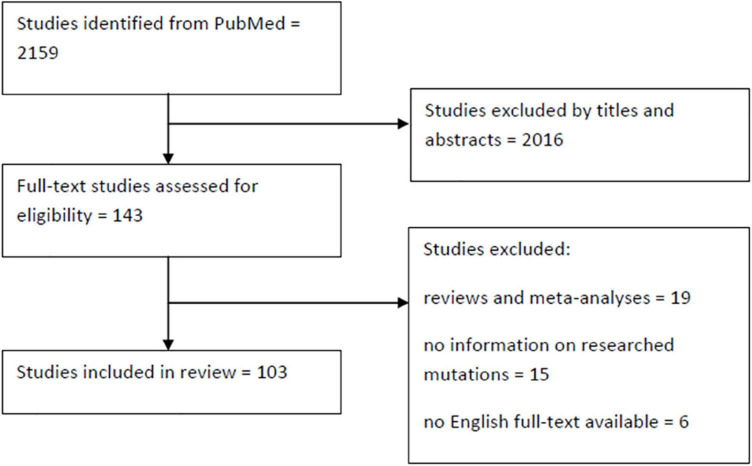
Flowchart illustrating the screening process.

**FIGURE 4:**
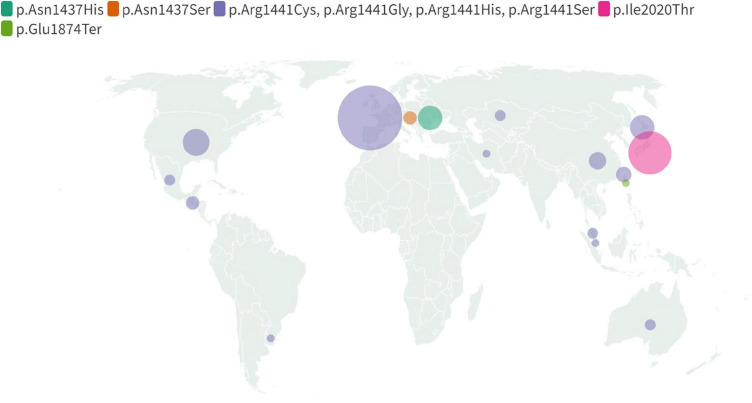
Map displaying global geographical distribution of the non-p.Gly2019Ser LRRK2 mutations. Due to limited data regarding carriers’ nationality and ethnicity following mutations were included: p.Asn1437His, p.Asn1437Ser, p.Ile2020Thr, p.Glu1874Ter and together indicated p. Arg1441Cys, p.Arg1441Gly, p.Arg1441His, p.Arg1441Ser. The map reflects information compiled in Supplementary Table 1.

**FIGURE 5:**
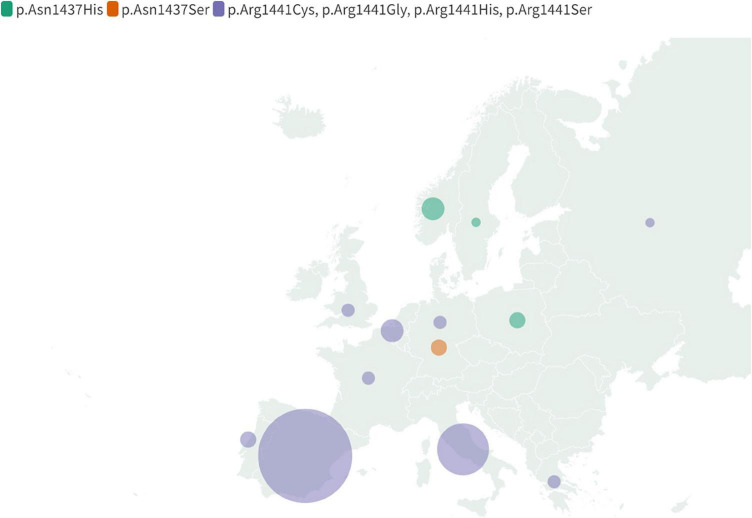
Map displaying regional distribution of the non-p.Gly2019Ser LRRK2 mutations in Europe. Following mutations were included: p.Asn1437His, p.Asn1437Ser and together indicated Arg1441Cys, p.Arg1441Gly, p.Arg1441His, p.Arg1441Ser. The map reflects information compiled in Supplementary Table 1.

References were identified through searches of PubMed using the following formula: [(LRRK2) OR (PARK8)] AND [(Parkinson’s Disease) OR (Parkinson)] AND [(mutation) OR (variant)] which provided us with 2,159 records. After initial analysis of titles and abstracts 143 papers were chosen for further assessment. The following were excluded: reviews and meta-analyses, publications concerning only p.Gly2019Ser or not concerning mutations reviewed here, papers without a full text and non-English texts. We also rejected studies that failed to find any carriers and in which direct sequencing of the mutations wasn’t performed. These usually concerned the whole *LRRK2* or most of its exons. We therefore identified 103 publications and described all non-p.Gly2019Ser mutations in LRRK2 that were reported as pathogenic in the ClinVar database (Figure 3). It is important to note that ACMG criteria were provided only for p.Arg1441Cys mutation, classifying it as pathogenic. Some other mutations (namely p.Asn1437Ser, p.Arg1441Gly, and p.Ile2020Thr) met other diagnostic assertion criteria, all of which were stated as pathogenic. The other mutations were not provided with any assertion criteria, mostly due to limited data surrounding them. The p.Asn1437Asp mutation figures as pathogenic in HGMD database. The results are summarized in Supplementary Table 1 and displayed in a form of map (Figures 4, 5).

## Results

### p.Asn1437His

The p.Asn1437His mutation was first identified in a large multi-generational Norwegian family. Further screening revealed an additional carrier affected by PD in the same population. It was proven to segregate with parkinsonism in autosomal dominant manner, but with reduced penetrance. Members of the affected families shared the same haplotype, suggesting the existence of a common ancestor (Aasly et al., 2010). Screening of 7 brains of PD patients from Sweden found one p.Asn1437His carrier with seemingly sporadic PD (Puschmann et al., 2012), though this variant was not found in a large study of the Swedish population, indicating its low prevalence (Puschmann et al., 2019).

#### Family presentation

**FIGURE 6:**
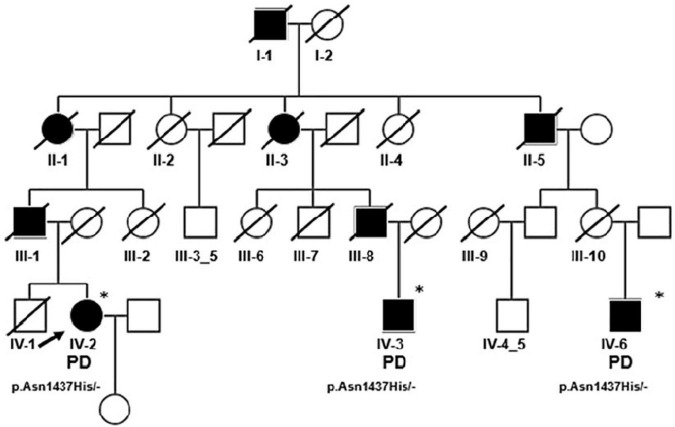
Pedigree of the PD/LRRK2 p.Asn1437His Polish Family; only generation IV was available for analysis, WES was performed to IV-2 (proband), mutation confirmation by Sanger sequencing was performed for proband’s cousins IV-3 I IV-6. *Indicates that the person was examined. Proband (IV-2) was a 63-year-old female with an 11-year history of PD. The first symptom was asymmetric left upper limb rigidity. After 2 years of disease levodopa treatment was initiated with good response. In neuropsychological examination only mild cognitive impairment was noted. The MRI was normal. When she was 59, she had deep brain stimulation electrodes (DBS) implanted to subthalamic nucleus (STN) with good response. Her cousin IV-3 was a 61-year-old male. The first symptom was right hand rigidity when 55 years old. Six months later levodopa treatment was implemented. He is currently still on a very low dose (300 mg total) of levodopa with good response. The youngest one (IV-6) developed symptoms of PD when he was 49. He presented left upper and later lower limb rigidity with subsequent left hand rest tremor. Three years after symptom onset levodopa treatment was initiated with good response. In the 5th year of the disease, the patient developed on-off fluctuations. He was treated with STN DBS when 57 years old. After the surgery he developed severe dysarthria and psychotic symptoms. However, after the dopamine agonist withdrawal and introduction of quetiapine treatment, the psychotic symptoms disappeared. He is now 64 years old, and the main symptoms are hypokinetic dysarthria with palilalia and echolalia. He is also suffering from severe gait disturbances with postural instability. He has also developed urinary incontinency. All were carriers of heterozygous pathogenic variant in the LRRK2 gene (NM_198578.3; GRCh38) c.[4309A > C];[-] causing missense mutation – p.[(Asn1437His)];[(-)].

A large PD family, 10 members affected over four generations, with an autosomal dominant pattern of inheritance was included in the analysis (Figure 6). General and neurological examinations of all affected, living family members were performed. Whole exome sequencing (WES; HiSeq Illumina platform) was performed for the proband and showed the presence (confirmed by Sanger sequencing) of the heterozygous variant in *LRRK2* NM_198578.3 (GRCh38) c.[4309A > C];[-] causing missense mutation – p.[(Asn1437His)];[(-)].

This is known mutation reported as pathogenic and PD causative (ClinVar ID39183; HGMD Professional 2021.4; CM107057).^1^

Because the cosegregation of mutation and phenotype (typical PD) was confirmed in the family, the p.Asn1437His was found to be a cause of disease in this pedigree. To our knowledge this is the first family with this mutation identified in Poland. Only a few such cases have been described globally, but detailed analysis of available data revealed similar symptoms in all patients—age of onset about 50 years old and asymmetric, slowly progressing parkinsonian symptoms (Aasly et al., 2010; Puschmann et al., 2012).

### p.Asn1437Ser and p.Asn1437Asp

The p.Asn1437Ser and p.Asn1437Asp substitutions are another pathogenic mutations in position 1437. A study of 70 non-consanguineous families of German descent affected by PD reported three carriers of p.Asn1437Ser mutation (Brockmann et al., 2011). All hailed from one family and exhibited symptoms of PD. p.Asn1437Ser was not found in any of the healthy subjects, further proving its pathogenicity. In another study of 240 Chinese from mainland China affected by PD (193 sporadic and 47 familial) one patient with patient with p.Asn1437Asp was detected (Li et al., 2020a). As the authors stated, it was a novel, potentially pathogenic mutation causative of PD.

### p.Arg1441Cys, p.Arg1441Gly, p.Arg1441His

There are 4 pathogenic mutations altering arginine in position 1441: p.Arg1441Cys, p.Arg1441Gly, p.Arg1441His, and p.Arg1441Ser. p.Arg1441Cys was identified for the first time by Zimprich et al. (2004) in a family from western Nebraska, USA, which was previously described by Wszolek et al. (2004). Zabetian et al. (2005) identified 1 carrier of p.Arg1441His and 1 carrier of p.Arg1441Cys mutations in a cohort of 371 American PD patients. Those mutations were absent in a control group. Only 2 additional studies reported codon 1441 mutations in North American PD patients. Latourelle et al. (2008) screened 903 PD patients, 58 unaffected members from 509 families, 126 randomly enrolled PD patients and 197 controls from North America for five pathogenic mutations, including p.Arg1441Cys and p.Arg1441His. They found 4 carriers of p.Arg1441Cys mutation among familial PD patients. The second study screened 956 PD patients from 430 multiplex pedigrees—two p.Arg1441Cys carriers were found (Pankratz et al., 2006).

European populations are among the most screened for p.Arg1441 mutations. Paisán-Ruíz et al. (2004) provided one of the first reports on mutations in codon 1441 among Europeans. They described mutation p.Arg1441Gly in 4 families of Basque ancestry and assessed its prevalence at 8%. Since then, Spain, especially the Basque population, has been thoroughly screened for LRRK2 mutations. Simón-Sánchez et al. (2006) conducted research to determine p.Arg1441Gly frequency in 158 Basque and 80 non-Basque PD patients, and they identified 17 mutation carriers (16 Basque and 1 non-Basque). They also estimated that p.Arg1441Gly accounts for 16.4% of familial and 4.0% of sporadic PD cases in the Basque population. Similar results were achieved by Gorostidi et al. (2009) in their study of 418 PD patients (218 Basque and 199 non-Basque habitants of Basque country) for the p.Arg1441Gly mutation. They identified 49 mutation carriers among patients of Basque origin and 6 among patients of non-Basque origin. González-Fernández et al. (2007) provided an analysis of 50 kindreds from the Basque region. In their cohort, p.Arg1441Gly presented in 10 families (21 individuals). Other studies covered populations from two regions near Basque country in northern Spain. Five p.Arg1441Gly mutation carriers were reported in a cohort of 225 patients from Asturias (Mata et al., 2005b,Infante et al., 2006). Few studies screening PD patients from other regions of Spain have been published until now. Two p.Arg1441Gly and 1 p.Arg1441Cys mutation carriers were identified in a Catalonian cohort of 302 patients and 3 p.Arg1441Gly mutation carriers were identified among PD patients from southern Spain (Gaig et al., 2006; Gao et al., 2009). The latter study revealed a compatible haplotype of p.Arg1441Gly carriers with patients of Basque origin. In a study by Bandrés-Ciga et al. (2016) the p.Arg1441Gly mutation was found in one familial and one sporadic PD patient from southern Spain. A study from central Spain found one p.Arg1441Cys carrier in a cohort of 117 patients with EOPD (Cristina et al., 2020).

Many southern European populations have been screened. Goldwurm et al. (2005) reported the presence of the p.Arg1441Cys mutation in 1 PD patient and the absence of the p.Arg1441Gly mutation in an Italian cohort including LOPD and EOPD patients. However, 4 p.Arg1441Cys and 1 p.Arg1441His carriers were discovered in an Italian cohort of 1,190 patients (1,088 with PD, 102 with other parkinsonism), and 13 PD patients from Campania harbored the p.Arg1441Cys mutation (Cilia et al., 2014; De Rosa et al., 2014). One carrier of the p.Arg1441His variant was found among 138 PD patients from Portugal (Ferreira et al., 2007). In another study of a Portuguese cohort by Zhang et al. (2013) p.Arg1441His was found in 2 of 61 PD patients. The two patients were from the same village, though a common ancestor within five generations was not found, providing evidence of a mutational hot-spot. As established later, all seven family members of those two probands carried this mutation. Three of them were unaffected and significantly younger, pointing to age-related penetrance. In a study of a Cretan population two carriers of p.Arg1441His were revealed (Spanaki et al., 2006). In a study of 356 PD patients and 208 controls from Sardinia two p.Arg1441Cys carriers were identified (Floris et al., 2009).

Gosal et al. (2007) provide a case report of an Irish patient affected by EOPD carrying p.Arg1441Cys variant. His deceased maternal aunt suffered from PD, but both of his parents (84 years old) and his four siblings (46–54 years old) were healthy. The carrier status of the family members is unknown. Two p.Arg1441Cys mutation carriers were found among EOPD patients from Germany (Hedrich et al., 2006). There were no mutation carriers among 103 French sporadic PD patients, though in a larger study of 182 French and 14 North African PD patients two p.Arg1441His cases were observed (Funalot et al., 2006; Lesage et al., 2009, 2020). Nuytemans et al. (2008) screened 304 Belgian PD patients (18.1% familial and 81.9% sporadic) and 278 controls for mutations in exons 29-31 and 38-44. They identified 6 carriers of the p.Arg1441Cys mutation that constituted 10.7% of familial PD cases. p.Arg1441Cys was also found in two patients from the UK (Tan et al., 2019). Pchelina et al. (2008) provided one of the first screenings for LRRK2 mutations in the Russian population and identified one p.Arg1441Cys sporadic PD carrier.

A few studies on the prevalence of p.Arg1441Cys/Gly/His mutations in South America have been conducted. A study by Cornejo-Olivas et al. (2017) discovered one p.Arg1441Cys mutation carrier in this population. This patient was affected by PD and reported a family history of the disease. Peruvian and Uruguayan PD patient populations were analyzed as well. Mata et al. (2009) screened 240 PD patients from Peru as well as 125 PD patients from Uruguay and found 1 patient with the p.Arg1441Gly mutation in the Uruguayan cohort. p.Arg1441Gly was also found in two patients from Peru and Uruguay (Cornejo-Olivas et al., 2017). Additional screening of five unaffected family members of the Peruvian p.Arg1441Gly patient revealed three more carriers. All family members shared the same haplotype as reported in p.Arg1441Gly carriers from the Basque region. Yescas et al. (2010) screened 319 PD patients and 200 controls from Mexico and found 2 carriers, one with the p.Arg1441Gly mutation and one with the p.Arg1441His.

In Asia, mutations in codon 1441 of LRRK2 are rare in comparison to other mutations. In a cohort of 384 LOPD patients from Singapore, one p.Arg1441Cys carrier was identified (Tan et al., 2006). One Iranian patient with the p.Arg1441Cys mutation was reported by Shojaee et al. (2009a). In a study of Kazakhstani PD patients no p.Arg1441His carriers were observed, though in a later study of this population carried out among EOPD patients p.Arg1441Cys mutation was found in two unrelated familial PD cases (Kaiyrzhanov et al., 2020, 2021). Two p.Arg1441Cys carriers were identified in a Chinese population in another study. The common founder effect was observed among the carriers (Chen et al., 2020). One Chinese patient was revealed to harbor the p.Arg1441His mutation (Zheng et al., 2020). Zhao et al. (2020) revealed two codon 1441 mutation carriers (p.Arg1441His and p.Arg1441Cys). Both probands had one family member unaffected by PD who carried the same mutation, suggesting reduced penetrance. Lin et al. (2008) screened 32 Taiwanese PD patients for the p.Arg1441His mutation and identified one carrier of this mutation. Wu et al. (2013) sequenced the whole coding region of *LRRK2* in a cohort comprised of 573 PD patients and 503 controls from Taiwan. One p.Arg1441His mutation carrier was reported. Lin et al. (2019) identified 2 carriers of the p.Arg1441His mutation in 571 Taiwanese PD patients. In a cohort of 499 Malaysian PD patients (165 had EOPD and 91 reported a family history of PD) p.Arg1441Cys was found in 2 sisters (0.4%) of Chinese ancestry (Lim et al., 2020). In a screening of a Japanese population 1 patient with p.Arg1441Gly was found. Additional familial analysis revealed two more affected carriers (mother and first cousin). They shared a haplotype different from that of the Spanish families (Hatano et al., 2014). Another large-sample study of a Japanese population presented 4 p.Arg1441Gly and 5 p.Arg1441His mutation carriers (Li et al., 2020b).

The only analysis of an Australian cohort was performed on a group of 830 PD patients. Two p.Arg1441His carriers were identified, though the authors weren’t able to identify their ancestral origin (Huang et al., 2007).

We found several studies concerning multinational populations. Mata et al. (2005a) sequenced all 51 exons of *LRRK2* and performed PCR in an affected proband from 100 multiplex families. They found the p.Arg1441Cys mutation in a North American family, the p.Arg1441His mutation in a Taiwanese family and the p.Arg1441Gly mutation in a Spanish family. Ross et al. (2011) screened a group of 8,611 sporadic PD patients of three ethnicities: Caucasian (6,995), Asian (1,376), and Arab-Berber (240) as well as 6,929 controls for the presence of LRRK2 mutations. The authors found 10 Caucasian carriers of p.Arg1441Cys and one Asian carrier of p.Arg1441His all of which were affected by PD. In an analysis of 60 families (50 from Italy, 9 from Brazil, and 1 from Portugal) two p.Arg1441Cys carriers were identified (Di Fonzo et al., 2006a). Lesage et al. (2009) analyzed all exons of *LRRK2* from 226 PD patients, mainly from France and north Africa. They identified 2 p.Arg1441His mutation carriers. In a research conducted by Deng et al. (2006) a Hispanic woman with the p.Arg1441Gly mutation was identified among 496 PD cases. p.Arg1441Cys was also reported in an Egyptian family with PD (Ali and Wszolek, 2022).

### p.Ile2020Thr

p.Ile2020Thr is a pathological mutation in exon 41 of *LRRK2* that rarely causes PD. It was reported only in members of Japanese families affected by PD compliable with autosomal-dominant inheritance and in one other family whose origin was not given by the authors (Zimprich et al., 2004; Funayama et al., 2005; Tomiyama et al., 2006). p.Ile2020Thr was shown to have a single-founder effect in Japanese patients (Tomiyama et al., 2006). As described below, all reported carriers were affected by PD except for three individuals without symptoms of parkinsonism, which however were within the variation of age at onset for the family (Funayama et al., 2005).

We found 40 papers regarding the p.Ile2020Thr mutation comprised of studies that examined the following populations: Asian (15), European (14), North American, African, international and unknown. The first cases of p.Ile2020Thr were described by Zimprich et al. (2004) who reported 3 members of one family affected by PD with typical age of onset (mean age of onset = 54 years). The authors didn’t provide enough information to determine the carriers’ ancestry.

p.Ile2020Thr was then reported in Japanese population as Funayama et al. (2005) sequenced the mutation in 25 members of a Japanese family named Sagamihara and its extended pedigree (19 patients affected by PD, 5 healthy members, and 1 spouse). p.Ile2020Thr was detected in all 19 affected members of the family and 3 unaffected members. Then the authors screened a larger Japanese cohort of sporadic PD patients and controls which failed to find any mutation carriers (Funayama et al., 2005). All of the Japanese cases described afterward shared a common founder effect with the Sagamihara family. Some authors reported that other Japanese p.Ile2020Thr carriers had the same haplotype within microsatellite markers and either them or their parent were born in or near the Sagamihara area (Tomiyama et al., 2006; Li et al., 2020b). The second study analyzed 1402 Japanese PD patients (749 with familial PD and 653 with sporadic PD) and 216 healthy controls. The mean age of onset was 51.4 for familial PD and 42.1 for sporadic PD cases. p.Ile2020Thr was found in 7 familial PD patients (Li et al., 2020b). The third study aimed to screen 904 parkin-negative PD patients (868 probands of whom 763 were Asians) from 18 countries across 5 continents for LRRK2 mutations. p.Ile2020Thr carriers were found among 3 Japanese patients of whom 2 were probands (the percentage of carriers in the cohort = 0.3%) (Tomiyama et al., 2006).

### Other rare mutations: p.Tyr1699Cys, p.Met1869Thr, p.Glu1874Ter, p.Ile1122Val

The p.Tyr1699Cys variant is a pathogenic mutation of *LRRK2* gene which is associated with PD. The mutation was detected in only one study. It was present in a German-Canadian family (8 PD individuals and 8 unaffected cases). All members with PD carried the mutation, though it was absent in healthy relatives. The authors also screened over 1,000 controls and 300 sporadic PD patients, but didn’t identify any p.Tyr1699Cys carriers (Zimprich et al., 2004).

The p.Met1869Thr mutation was found in two studies. One of them assessed a multinational cohort of PD patients who had familial PD. The authors analyzed all *LRRK2* exons in 100 probands. p.Met1869Thr was found in one North American patient of Caucasian ethnicity whose affected sibling didn’t carry the mutation (Mata et al., 2005a). Ross et al. (2011) reported a group of 7 carriers of p.Met1869Thr consisting of 5 patients affected by PD and 2 healthy controls. All of the subjects were found among Caucasian cohort.

p.Glu1874Ter was reported by Di Fonzo et al. (2006b) who studied 592 idiopathic PD Taiwanese patients and 370 healthy controls. The mutation was found in one case of PD (0.001%). p.Glu1874Ter is therefore of marginal importance in the pathogenesis of monogenetic PD taking into consideration the lack of information about the mutation and the fact that it has not been reported by any other study.

p.Ile1122Val constitutes a rare pathogenic mutation causing PD which was identified by Zimprich et al. (2004) while genotyping 44 PD families. Up until now, no other study has described the mutation.

## Discussion

Our report summarizes the prevalence of LRRK2 non-p.Gly2019Ser pathogenic mutations in the world population and presents the first case of an extremely rare LRRK2 p.Asn1437His mutation in a Polish PD family.

To this day p.Asn1437His has only been discovered in two Norwegian families and one Swedish patient (Supplementary Table 1). Age of onset in all cases was approximately 50 years, they all exhibited asymmetrical onset of PD with slow progression and all had a good response to levodopa. Four patients in total were treated with STN DBS-two with good response. What is more, some PD carriers of p.Asn1437His exhibited a more severe phenotype with psychotic symptoms and dysarthria (Polish patient), or severe depression (Swedish patient) (Aasly et al., 2010; Puschmann et al., 2012). However, no clear differences in phenotypes were observed in comparison to sporadic PD cases, which is in line with other findings regarding LRRK2 mutation-associated PD. In order to assess the possibility that the more severe phenotype of PD is indeed linked with p.Asn1437His more research should be performed.

LRRK2 is a large multi-domain protein consisting of 7 putative domains, including the Ras-of-complex proteins (ROC) GTPase domain, the C-terminal of ROC (COR) domain and the kinase (KIN) domain. Kinase and GTPase activities are performed respectively by KIN and ROC. This dual enzymatic activity suggests that the KIN and ROC domains affect each other, though the exact mechanism remains unknown. Disease pathogenesis is associated with elevated kinase activity. p.Asn1437His mutation is located in the ROC-COR region. It promotes the formation of stable ROC domain homodimer, thus impairing its GTPase activity. Moreover, ROC_p_._Asn1437His_ has reduced GTP binding affinity and a slower GTP dissociation rate. The lowered GTPase activity elevates kinase activity, explaining the pathogenicity of the p.Asn1437His mutation (Huang et al., 2019).

As many clinical trials concerning specific types of PD are emerging, we consider it crucial to summarize the state-of-the-art knowledge on *LRRK2* mutations, their pathogenicity and prevalence, which is complex. We focused on non-p.Gly2019Ser LRRK2 mutations because they were less frequent analyzed. However, since the introduction of high-throughput gene sequencing methods, more and more data are suggesting that the mutations in LRRK2 are rather rare and have varied distributions in different populations. It is therefore important to consider their prevalence in specific cohorts, especially if this data were to be used in clinical trials. Over the past quarter century there has been an enormous increase in our understanding of the genetics behind PD and its pathophysiology (Cherian and Divya, 2020; Sjögren et al., 2021). Autosomal dominant (*LRRK2, SNCA, VPS35*) and autosomal recessive (*PINK1, DJ-1, Parkin*) genes are found in about 5-10% of all patients suffering from PD. Furthermore, genetic studies revealed several risk factors, including the most frequent one—*GBA*, or specific *LRRK2* mutations such as p.Gly2385Arg and p.Arg1628Pro.

Among pathogenic mutations, *LRRK2* is the most common cause of inherited PD. It has been described mainly in European populations, but more and more information concerning other cohorts or ethnicities is appearing. All this leads to such possible future perspectives as creation of agents targeting a specific gene or even mutation—which could be neuroprotective or used as a biomarker. Currently, more than 140 human studies concerning PD are underway (Sjögren et al., 2021). They include α-synuclein, GBA and LRRK2 targeting treatments, with the use of antibodies, vaccines, gene therapies and small-molecule compounds.

## Conclusion

We herein report the first case of a Polish PD family carrying p.Asn1437His which is one of the pathogenic mutations causing PD. The mutation’s pathogenicity is due to its impact on GTPase and kinase activity of LRRK2. Up until now, it has been reported only in Norwegian and Swedish patients. All the carriers exhibited similar symptoms and age of onset of PD as non-LRRK2 linked PD cases, although some of them had a more severe phenotype.

Recently, more findings concerning rarity, distribution and pathogenicity of non-p.Gly2019Ser LRRK2 mutations have emerged. In addition to the aforementioned mutation, p.Arg1441Cys, p.Arg1441Gly and p.Arg1441His were reported in European (mainly Hispanic) populations, though few patients were found in Asian and South American cohorts. p.Ile2020Thr was found only in Japanese patients. They all shared a common founder effect with the first family described carrying the mutation (the Sagamihara family). p.Met1869Thr was described uniquely in Caucasians. However, some of the carriers were unaffected by PD. p.Tyr1699Cys, p.Ile1122Val, Glu1874Ter are rare mutations that were reported by one study each.

The factors mentioned above should be taken into consideration, especially in clinical trials. The ground-breaking progress in our knowledge of PD’s genetics and pathophysiology potentially enables the creation of better diagnostic and treatment methods.

## Summary

1. We report the first case of a Polish PD family carrying p.Asn1437His previously found only in Norwegian and Swedish patients.
2. p.Arg1441Cys, p.Arg1441Gly, p.Arg1441His occur mostly in Hispanic and to a smaller degree in European populations.
3. p.Ile2020Thr concerns uniquely Japanese patients.
4. p.Tyr1699Cys, p.Met1869Thr, p.Ile1122Val, Glu1874Ter are rarely reported mutations.
5. The factors such as distribution and pathogenicity of non-p.Gly2019Ser LRRK2 mutations should be taken into consideration.

## Author contributions

ŁM: conceptualization, supervision, and materials. PT, IC, PS, and PP: writing – original draft preparation. ŁM, MF, DH-Z, DK, PT and IC: writing –review and editing. SS, JS, ŁM, and DK: clinical examination. DH-Z: experiments conducing. All authors have read and agreed to the published version of the manuscript.

## Footnotes

1
https://my.qiagendigitalinsights.com/bbp/view/hgmd/pro
