# Supplementary material for: Review of the epidemiology and variability of LRRK2 non-p.Gly2019Ser pathogenic mutations in Parkinson’s disease
Source: Front Neurosci. 2022 Sep 20;16:971270. doi: 10.3389/fnins.2022.971270 (PMC9530194; doi:10.3389/fnins.2022.971270)
Supplement: Supplementary file 1 [file Table_1.docx]

**Table 1.** Summary of the LRRK2 pathogenic mutations’ prevalence in different world populations. EOPD – early onset Parkinson Disease, LOPD – late onset Parkinson Disease, ADPD – autosomal dominant Parkinson Disease, ARPD – autosomal recessive Parkinson Disease, ND – no data, *  – indicates inhabitants of Taiwan

| Mutation | Population | PD patients | Control  group | Mutation carriers (cases/ controls) | Specific features | Reference |
| --- | --- | --- | --- | --- | --- | --- |
| p.Asn1437His | Norwegian | 699 | 627 | 6/0 | 7 affected and 4 unaffected members enrolled | Aasly et al. (2010) |
|  | Caucasian | 6995 | 5595 | 0/0 |  | Ross et al. (2011) |
|  | Asian | 1376 | 962 | 0/0 |  |  |
|  | Arab-Berber | 240 | 372 | 0/0 |  |  |
|  | Swedish | 7 | 0 | 1/- |  | Puschmann et al. (2012) |
|  | Greek | 48 | 0 | 0/- |  | Bozi et al. (2014) |
|  | Swedish | 2206 | 0 | 0/- |  | Puschmann et al. (2019) |
|  | Kazakhstani | 240 | 199 | 0/0 | Different ethnicities were enrolled similar to country’s percentage of population | Kaiyrzhanov et al. (2020) |
|  | Nigerian | 92 | 210 | 0/0 |  | Rizig et al. (2021) |
| p.Asn1437Ser | German |  |  | 3/0 | 70 families were assessed. All carriers hailed from one family and were affected by PD. | Brockmann et al. (2011) |
| p.Ile1122Val | Caucasian | 44 | 0 | 2/- | One patient per PD family was chosen.  Carriers were members of one family. | Zimprich et al. (2004) |
|  | Japanese | 203 | 190 | 0/0 | 19 affected and 6 unaffected members of the same family were enrolled. | Funayama et al. (2005) |
|  | American | 504 | 314 | 0/0 |  | Clark et al. (2006) |
|  | Spanish | 276 | 0 | 0/0 | Population consisted of 66.4% Basques. | Simón-Sánchez et al. (2006) |
|  | Han Chinese, Malay, Indian | 458 | 0 | 0/- | 74 patients were affected by other parkinsonism | Tan et al. (2006) |
|  | Canadian | 230 | 129 | 0/0 |  | Grimes et al. (2007) |
|  | Austrian | 192 | 288 | 0/0 |  | Haubenberger et al. (2007) |
|  | Brazilian | 72 | 81 | 0/0 |  | Aguiar Pde et al. (2008) |
|  | Brazilian | 119 | 0 | 0/- |  | Barsottini et al. (2009) |
|  | Caucasian | 6995 | 5595 | 0/0 |  | Ross et al. (2011) |
|  | Asian | 1376 | 962 | 0/0 |  |  |
|  | Arab-Berber | 240 | 372 | 0/0 |  |  |
|  | Han Chinese* | 626 | 473 | 0/0 |  | Wu-Chou et al. (2013) |
|  | Italian | 88 | 0 | 0/- |  | Anfossi et al. (2014) |
|  | Nigerian | 92 | 210 | 0/0 |  | Rizig et al. (2021) |
| p.Arg1441Cys, p.Arg1441Gly, p.Arg1441His, p.Arg1441Ser | ND | 44 | 0 | 2/- | All patients had familial PD.  The carriers were members of one family. | Zimprich et al. (2004) |
|  | Norwegian | 435 | 519 | 0/0 |  | Aasly et al. (2005) |
|  | American | 818 | 1322 | 0/0 | The patient cohort comprised 32 affected siblings.  The controls consisted of 1044 unaffected siblings. | Farrer et al. (2005) |
|  | Italian | 662 | 440 | 1/0 | The patients comprised 230 EOPD cases and 33 affected relatives.  The carrier had p.Arg1441Cys. | Goldwurm et al. (2005) |
|  | Caucasian, Asian | 100 | 300 | 12/0 | The patient cohort comprised only familial cases of PD.  3 carriers of p.Arg1441Cys, 7 carriers of p.Arg1441Gly, and 2 carriers of p.Arg1441His were found. | Mata et al. (2005a) |
|  | Spanish | 225 | 100 | 5/0 | Only p.Arg1441Gly was established.  All the carriers had p.Arg1441Gly. | Mata et al. (2005b) |
|  | American | 371 | 281 | 2/0 | 1 carrier of p.Arg1441His, and 1 carrier of p.Arg1441Cys were found. | Zabetian et al. (2005) |
|  | Swedish | 284 | 305 | 0/0 |  | Carmine Belin et al. (2006) |
|  | North American | 504 | 314 | 0/0 |  | Clark et al. (2006) |
|  | North American | 496 | 220 | 1/0 | One patient carried p.Arg1441Gly and was of Hispanic origin.  Population consisted of ethnicities: Caucasians, Ashkenazi Jews, and Hispanic | Deng et al. (2006) |
|  | Italian, Brazilian, Portuguese | 60 | 0 | 2/- | Carriers were Italians. | Di Fonzo et al. (2006a) |
|  | French | 103 | 0 | 0/- | Only p.Arg1441Gly was established. | Funalot et al. (2006) |
|  | Spanish  (Catalonian) | 302 | 0 | 3/- | 2 p.Arg1441Gly and 1 p.Arg1441Cys carriers were found. | Gaig et al. (2006) |
|  | German, Serbian, Italian | 360 | 100 | 2/0 | Patients consisted of 318 EOPD.  2 p.Arg1441Cys carriers were found. | Hedrich et al. (2006) |
|  | Spanish  (Cantabrian) | 105 | 310 | 0/0 | Only p.Arg1441Gly was established. | Infante et al. (2006) |
|  | North American | 956 | 0 | 2/- | Both carriers had p.Arg1441Cys and were brothers. | Pankratz et al. (2006) |
|  | Indian | 800 | 212 | 0/0 |  | Punia et al. (2006) |
|  | German | 120 | 0 | 0/- | 10 patients were defined as EOPD | Schlitter et al. (2006) |
|  | American | 202 | 109 | 0/0 | Only p.Arg1441Cys and p.Arg1441Gly were established. | Scholz et al. (2006) |
|  | Basque | 238 | 0 | 17/- | Only p.Arg1441Gly was established.  The patient cohort comprised 86 familial cases.  Among carriers 7 were familial PD patients. | Simón-Sánchez et al. (2006) |
|  | Cretan | 266 | 300 | 2/0 | Carriers had p.Arg1441His. | Spanaki et al. (2006) |
|  | Singaporean | 384 | 0 | 1/- | The carrier had p.Arg1441Cys. | Tan et al. (2006) |
|  | Sardinian | 98 | 55 | 0/0 |  | Cossu et al. (2007) |
|  | French-Canadian | 125 | 95 | 0/0 |  | Dupré et al. (2007) |
|  | Portuguese | 138 | 101 | 1/0 | The carrier had p.Arg1441His. | Ferreira et al. (2007) |
|  | Spanish (Basque) | 6 | 98 | 15/6 | The patient cohort consisted of 61 familial cases of PD from 50 families.  The controls comprised 30 unaffected relatives and 68 unrelated subjects.  Only p.Arg1441Gly was established.  All of carriers were had familial PD. | González-Fernández et al. (2007) |
|  | Canadian | 230 | 0 | 0/- |  | Grimes et al. (2007) |
|  | Austrian | 162 | 288 | 0/0 |  | Haubenberger et al. (2007) |
|  | Australian | 830 | 0 | 2/- | Both of the carriers had p.Arg1441His. | Huang et al. (2007) |
|  | Jewish Israeli | 472 | 252 | 0/0 | The patients comprised 342 Ashkenazi Jews, 112 non-Ashkenazi Israelis, and 14 mixed cases. | Orr-Urtreger et al. (2007) |
|  | Chilean | 166 | 153 | 0/0 |  | Perez-Pastene et al. (2007) |
|  | Brazilian | 72 | 81 | 0/0 | Only p.Arg1441Cys was established.  The affected cases were defined as EOPD. | Aguiar Pde et al. (2008) |
|  | South Korean | 72 | 0 | 0/- | The affected cases were defined as EOPD. | Choi et al. (2008) |
|  | North American | 1029 | 197 | 4/0 | Only p.Arg1441Cys and p.Arg1441His were established.  All of the carriers had p.Arg1441Cys. | Latourelle et al. (2008) |
|  | Han Chinese* | 32 | 0 | 1/- | Only p.Arg1441His was established. | Lin et al. (2008) |
|  | German | 1049 | 0 | 0/- | Among affected cases there were 268 EOPD patients. | Möller et al. (2008) |
|  | Belgian | 304 | 278 | 6/0 |  | Nuytemans et al. (2008) |
|  | Nigerian | 57 | 51 | 0/0 |  | Okubadejo et al. (2008) |
|  | Russian | 274 | 100 | 1/0 | The carrier had p.Arg1441Cys. | Pchelina et al. (2008) |
|  | Brazilian | 154 | 250 | 0/0 |  | Pimentel et al. (2008) |
|  | Brazilian | 119 | 0 | 0/- | Only p.Arg1441Cys was established. | Barsottini et al. (2009) |
|  | Sardinian | 356 | 208 | 2/0 | Both of the carriers had p.Arg1441Cys. | Floris et al. (2009) |
|  | Southern Spanish | 187 | 287 | 3/0 | All of the carriers had p.Arg1441Gly. | Gao et al. (2009) |
|  | Basque | 418 | 138 | 55/0 | Only p.Arg1441Gly was established.  199 affected cases were of non-Basque origin.  Among carriers 49 were Basque and 6 were non-Basque. | Gorostidi et al. (2009) |
|  | French, North African | 226 | 174 | 2/0 | All patients had familial PD.  182 patients were French, 14 North African, and 6 Caribbean.  The controls were Europeans.  Both the carriers had p.Arg1441His. | Lesage et al. (2009) |
|  | Dutch | 187 | 375 | 0/0 | All patients were defined as EOPD. | Macedo et al. (2009) |
|  | Peruvian | 240 | 0 | 0/- | Only p.Arg1441Cys and p.Arg1441Gly were established. | Mata et al. (2009) |
|  | Uruguayan | 125 | 0 | 1/- | Only p.Arg1441Cys and p.Arg1441Gly were established.  The carrier had p.Arg141Gly. | Mata et al. (2009) |
|  | Iranian | 205 | 200 | 1/0 | The carrier had p.Arg1441Cys. | Shojaee et al. (2009b) |
|  | North American | 953 | 0 | 0/- | The population comprised diverse ancestries. | Alcalay et al. (2010) |
|  | East Indian | 150 | 170 | 0/0 |  | Sanyal et al. (2010) |
|  | Mexican | 319 | 200 | 2/0 | One p.Arg1441Gly carrier and 1 p.Arg1441His carrier were found. | Yescas et al. (2010) |
|  | Italian (Campania) | 192 | 0 | 8/- | All of the carriers had p.Arg1441Cys. | Criscuolo et al. (2011) |
|  | Han Chinese | 221 | 120 | 0/0 |  | Hu et al. (2011) |
|  | Turkish | 255 | 0 | 0/- |  | Hanagasi et al. (2011) |
|  | Caucasian | 6995 | 5595 | 10/0 | All of the carriers had p.Arg1441Cys. | Ross et al. (2011) |
|  | Asian | 1376 | 962 | 1/0 | The carrier had p.Arg1441His. |  |
|  | Arab-Berber | 240 | 372 | 0/0 |  |  |
|  | Ghanaian | 54 | 46 | 0/0 |  | Cilia et al. (2012) |
|  | Indian | 300 | 446 | 0/0 |  | Sadhukhan et al. (2012) |
|  | Slovak | 216 | 0 | 0/- |  | Bognar et al. (2013) |
|  | Han Chinese* | 573 | 503 | 1/0 | The carrier had p.Arg1441His. | Wu et al. (2013) |
|  | Han Chinese* | 626 | 473 | 0/0 |  | Wu-Chou et al. (2013) |
|  | Portuguese | 312 | 138 | 2/0 | Only p.Arg1441His was assessed in the control group.  Both of the carriers had p.Arg1441His. | Zhang et al. (2013) |
|  | Italian (Calabrian) | 88 | 0 | 0/- |  | Anfossi et al. (2014) |
|  | Greek | 48 | 0 | 0/- |  | Bozi et al. (2014) |
|  | Italian | 1190 | 0 | 5/- | The patients comprised of 102 cases defined as other parkinsonism.  Among the carriers 4 had p.Arg1441Cys, 1 had p.Arg1441His. | Cilia et al. (2014) |
|  | Italian,  Campanian | 513 | 0 | 13/- | All of the carriers had p.Arg1441Cys. | De Rosa et al. (2014) |
|  | Japanese | 871 | 0 | 1 | The carrier had p.Arg1441Gly. | Hatano et al. (2014) |
|  | Serbian | 486 | 143 | 0/0 |  | Janković et al. (2015) |
|  | Han and Uyghur Chinese | 312 | 359 | 0/0 |  | Li et al. (2015) |
|  | Spanish | 134 | 0 | 2/0 | Both of the carriers had p.Arg1441Gly. | Bandrés-Ciga et al. (2016) |
|  | American | 3 | - | 3/0 | The patients were members of one family.  All of them carried p.Arg1441Ser. | Mata et al. (2016) |
|  | Maltese | 148 | 0 | 0/0 | Only p.Arg1441Gly was established. | Zahra et al. (2016) |
|  | Latin American | 1734 | 1102 | 3/0 | One p.Arg1441Cys carrier and 2 p.Arg1441Gly carriers were found. | Cornejo-Olivas et al. (2017) |
|  | Finnish | 852 | 403 | 0/0 |  | Ylönen et al. (2017) |
|  | Spanish | 4783 | 3066 | 1/0 | The carrier had p.Arg1441Gly. | Bandres-Ciga et al. (2019) |
|  | Han Chinese* | 571 | 0 | 2/- | The patients comprised of 304 EOPD cases and 247 individuals with familial PD.  Two familial PD patients carried p.Arg1441His. | Lin et al. (2019) |
|  | Swedish | 2206 | 0 | 0/- | Only p.Arg1441His was established.  12.1% of patients had familial PD. | Puschmann et al. (2019) |
|  | British | 2003 | 0 | 2/- | 96.3% were Caucasians.  408 patients were defined as EOPD. | Tan et al. (2019) |
|  | Han Chinese | 662 | 0 | 2/- | All of patients were defined as EOPD, 144 of them had familial PD.  Both of the carriers had p.Arg1441Cys. | Chen et al. (2020) |
|  | Spanish | 117 | 0 | 1/- | All patients were defined as EOPD, 33 of them had familial PD.  The carrier had p.Arg1441Cys. | Cristina et al. (2020) |
|  | Kazakhstani | 264 | 200 | 0/0 | Only p.Arg1441His was established. | Kaiyrzhanov et al. (2020) |
|  | Japanese | 1402 | 216 | 9/0 | 749 patients had familial PD.  Four carriers of p.Arg1441Gly and 5 carriers of p.Arg1441His were found. | Li et al. (2020b) |
|  | Malay | 499 | 0 | 2/- | 165 patients had EOPD, 91 had familial PD.  The carriers were sisters of Chinese origin. | Lim et al. (2020) |
|  | Han Chinese | 1676 | 1279 | 2/4 | All of the patients were unrelated. 434 of them had familial PD (192 ARPD, 242 ADPD) and 1242 were sporadic EOPD cases.  All of the affected carriers had ADPD and they carried p.Arg1441Cys and p.Arg1441His.  Two of unaffected carriers had p.Arg1441Cys and 2 had p.Arg1441His. | Zhao et al. (2020) |
|  | Han Chinese | 191 | 200 | 1/0 | The patients comprised of 106 EOPD.  The carrier had p.Arg1441His. | Zheng et al. (2020) |
|  | Kazakhstani | 50 | 0 | 2/- | All of the patients were defined as EOPD and 10 had familial PD.  Both of the carriers had p.Arg1441Cys. | Kaiyrzhanov et al. (2021) |
|  | Nigerian | 92 | 210 | 0/0 |  | Rizig et al. (2021) |
| p.Ile2020Thr | Norwegian | 435 | 519 | 0/0 |  | Aasly et al. (2005) |
|  | Polish | 174 | 190 | 0/0 | 21 patients had familial PD. | Bialecka et al. (2005) |
|  | American | 818 | 1322 | 0/0 | The patients comprised 32 affected siblings while the controls consisted of 1044 unaffected siblings. | Farrer et al. (2005) |
|  | Japanese | 213 | 184 | 22/0 | Except for sporadic cases one big PD family was included (the Sagamihara family: 19 affected members, 5 healthy, and 1 spouse).  All of the mutation carriers were members of the family. | Funayama et al. (2005) |
|  | Han Chinese* | 624 | 0 | 0/- |  | Lu et al. (2005) |
|  | Swedish | 284 | 305 | 0/- | 42 patients had familial PD. | Carmine Belin et al. (2006) |
|  | North American | 965 | 0 | 0/- | The population consisted of 94% Caucasian and 6% Hispanic ethnicities.  All of the patients had familial PD. | Pankratz et al. (2006) |
|  | Indian | 748 | 0 | 0/- |  | Punia et al. (2006) |
|  | Spanish | 276 | 0 | 0/- | 158 Basques were enrolled.  86 patients had familial PD | Simón-Sánchez et al. (2006) |
|  | Cretan | 266 | 300 | 0/0 | 92 patients had familial PD. | Spanaki et al. (2006) |
|  | Multinational | 458 | 0 | 0/- | 85% of population were Han Chinese, 9% Malaysian, 6% Indian.  74 patients had other parkinsonism. | Tan et al. (2006) |
|  | Multinational | 868 | ND | 2/- | 210 patients had familial PD.  All of the carriers were Japanese and shared a common ancestor with the Sagamihara family. | Tomiyama et al. (2006) |
|  | French-Canadian | 125 | 95 | 0/0 | The patients comprised 11 EOPD and 28 cases with familial PD. | Dupré et al. (2007) |
|  | Canadian | 230 | 129 | 0/0 | 34% patients had familial PD. | Grimes et al. (2007) |
|  | Austrian | 192 | 288 | 0/0 | The patients comprised 162 LOPD and 30 cases with dementia with Lewy bodies. | Haubenberger et al. (2007) |
|  | Jewish-Israeli | 472 | 1802 | 0/0 | The patients comprise 344 Ashkenazi descents and 131 had familial PD. | Orr-Urtreger et al. (2007) |
|  | Multinational | 776 | 155 | 0/0 | The patients comprised 650 cases with familial PD. | Latourelle et al. (2008) |
|  | Han Chinese* | 32 | 0 | 0/- | All of the patients had familial PD. | Lin et al. (2008) |
|  | Russian | 274 | 100 | 0/0 | The patients comprised 85 cases with familial PD. | Pchelina et al. (2008) |
|  | Brazilian | 154 | 250 | 0/0 | 34% of the patients had EOPD and 23 had familial PD. | Pimentel et al. (2008) |
|  | Sardinian | 356 | 208 | 0/0 | The patients comprised 28 EOPD and 65 familial PD cases. | Floris et al. (2009) |
|  | Japanese | 631 | 320 | 0/0 |  | Zabetian et al. (2009) |
|  | American | 953 | 0 | 0/- | Only EOPD patients were enrolled: 139 were Jewish and 77 were Hispanic. | Alcalay et al. (2010) |
|  | East Indian | 150 | 170 | 0/0 | 9 patients had familial PD. | Sanyal et al. (2010) |
|  | Mexican-mestizos | 319 | 200 | 0/0 | 67 patients had familial PD. | Yescas et al. (2010) |
|  | Turkish | 255 | 0 | 0/- | 110 patients had familial PD. | Hanagasi et al. (2011) |
|  | Han Chinese | 221 | 120 | 0/0 |  | Hu et al. (2011) |
|  | Ghanian | 54 | 46 | 0/0 | The patients comprised 10 familial PD cases. | Cilia et al. (2012) |
|  | UK | 136 | 0 | 0/- | Only EOPD patients were enrolled. | Kilarski et al. (2012) |
|  | East Indian | 300 | 446 | 0/0 |  | Sadhukhan et al. (2012) |
|  | Slovak | 216 | 0 | 0/- | 39 EOPD cases were enrolled. | Bognar et al. (2013) |
|  | Italian Calabrian | 88 | 200 | 0/0 | The patients comprised 25 familial PD cases. | Anfossi et al. (2014) |
|  | Greek | 48 | 0 | 0/- | 12 EOPD were enrolled. | Bozi et al. (2014) |
|  | Italian | 2523 | 0 | 0/- | The patients comprised 331 other parkinsonism cases. | Cilia et al. (2014) |
|  | Japanese | 871 | 0 | 0/- | 441 patients had familial PD. | Hatano et al. (2014) |
|  | Swedish | 2206 | 0 | 0/- |  | Puschmann et al. (2019) |
|  | Kazakhstani | 246 | 200 | 0/0 |  | Kaiyrzhanov et al. (2020) |
|  | Japanese | 1402 | 216 | 7/0 | The patients comprised 749 familial PD cases.  The carriers shared common ancestor with the Sagamihara family. | Li et al. (2020b) |
|  | Nigerian | 92 | 210 | 0/0 |  | Rizig et al. (2021) |
|  | Multinational | 8611 | 6929 | 0/0 |  | Ross et al. (2011) |
| p.Tyr1699Cys | ND | 308 | 1008 | 8/0 | 8 affected and 8 unaffected members of one German-Canadian family were enrolled.  All of the carriers were part of the family. | Zimprich et al. (2004) |
|  | Norwegian | 435 | 519 | 0/0 |  | Aasly et al. (2005) |
|  | Slovak | 216 | 0 | 0/- |  | Bognar et al. (2013) |
|  | Greek | 81 | 0 | 0/- |  | Bozi et al. (2014) |
|  | Japanese | 188 | 184 | 0/0 |  | Funayama et al. (2005) |
|  | Italian | 600 | 440 | 0/0 |  | Goldwurm et al. (2005) |
|  | Canadian | 230 | 129 | 0/0 |  | Grimes et al. (2007) |
|  | Turkish | 255 | 0 | 0/- |  | Hanagasi et al. (2011) |
|  | Austrian | 146 | 288 | 0/0 |  | Haubenberger et al. (2007) |
|  | Kazakhstani | 246 | 200 | 0/0 |  | Kaiyrzhanov et al. (2020) |
|  | Caucasian | 956 | 0 | 0/- |  | Pankratz et al. (2006) |
|  | Swedish | 2206 | 0 | 0/- |  | Puschmann et al. (2019) |
|  | Caucasian | 6995 | 5595 | 0/0 |  | Ross et al. (2011) |
|  | Asian | 1376 | 962 | 0/0 |  |  |
|  | Arab-Berber | 240 | 372 | 0/0 |  |  |
|  | Indian | 300 | 446 | 0/0 |  | Sadhukhan et al. (2012) |
|  | Indian | 150 | 170 | 0/0 |  | Sanyal et al. (2010) |
|  | Iranian | 205 | 200 | 0/0 |  | Shojaee et al. (2009b) |
|  | Han Chinese* | 626 | 473 | 0/0 |  | Wu-Chou et al. (2013) |
|  | Mexican | 319 | 200 | 0/0 |  | Yescas et al. (2010) |
| p.Met1869Thr | Multinational | 100 | 1000 | 1/0 | All of the patients enrolled were probands with a family history of PD.  The affected sibling of the same family as the carrier didn’t have p.Met1869Thr. | Mata et al. (2005a) |
|  | Han Chinese* | 608 | 373 | 0/0 |  | Di Fonzo et al. (2006b) |
|  | Canadian | 230 | 129 | 0/0 |  | Grimes et al. (2007) |
|  | Austrian | 146 | 288 | 0/0 |  | Haubenberger et al. (2007) |
|  | Caucasian | 956 | 0 | 0/- |  | Pankratz et al. (2006) |
|  | Caucasian | 6995 | 5595 | 5/2 |  | Ross et al. (2011) |
|  | Asian | 1376 | 962 | 0/0 |  |  |
|  | Arab-Berber | 240 | 372 | 0/0 |  |  |
|  | Han Chinese* | 626 | 473 | 0/0 |  | Wu-Chou et al. (2013) |
| p.Glu1874Ter | Han Chinese* | 593 | 370 | 1/0 |  | Di Fonzo et al. (2006b) |
|  | Multinational | 8611 | 6929 | 0/0 |  | Ross et al. (2011) |
